# *In crystallo* optical spectroscopy (*ic*OS) as a complementary tool on the macromolecular crystallography beamlines of the ESRF

**DOI:** 10.1107/S139900471401517X

**Published:** 2015-01-01

**Authors:** David von Stetten, Thierry Giraud, Philippe Carpentier, Franc Sever, Maxime Terrien, Fabien Dobias, Douglas H. Juers, David Flot, Christoph Mueller-Dieckmann, Gordon A. Leonard, Daniele de Sanctis, Antoine Royant

**Affiliations:** a European Synchrotron Radiation Facility, F-38043 Grenoble, France; b Université Grenoble Alpes, IBS, F-38044 Grenoble, France; c CNRS, IBS, F-38044 Grenoble, France; d CEA, IBS, F-38044 Grenoble, France; e Department of Physics, Whitman College, Walla Walla, WA 99362, USA

**Keywords:** *in crystallo* optical spectroscopy, Cryobench, UV-visible light absorption spectroscopy, fluorescence spectroscopy, Raman spectroscopy

## Abstract

The analysis of structural data obtained by X-ray crystallo­graphy benefits from information obtained from complementary techniques, especially as applied to the crystals themselves. As a consequence, optical spectroscopies in structural biology have become instrumental in assessing the relevance and context of many crystallographic results. Since the year 2000, it has been possible to record such data adjacent to, or directly on, the Structural Biology Group beamlines of the ESRF. A core laboratory featuring various spectrometers, named the Cryobench, is now in its third version and houses portable devices that can be directly mounted on beamlines. This paper reports the current status of the Cryobench, which is now located on the MAD beamline ID29 and is thus called the ID29S-Cryobench (where S stands for ‘spectroscopy’). It also reviews the diverse experiments that can be performed at the Cryobench, highlighting the various scientific questions that can be addressed.

## Abstract 2

The current version of the Cryobench *in crystallo* optical spectroscopy facility of the ESRF is presented. The diverse experiments that can be performed at the Cryobench are also reviewed.

## 1. Introduction   

Performing optical spectroscopy on crystals, or *in crystallo* optical spectroscopy (*ic*OS), has progressively matured as a technique complementary to protein crystallography. Originally developed for Laue diffraction experiments on coloured proteins such as myoglobin or photoactive yellow protein, instruments and facilities have been progressively developed over the last 20 years. The first microspectrophotometers (microspecs) capable of recording UV–visible light absorption (UV–vis absorption) spectra on protein crystals that can be orientated in three dimensions were designed in the early 1990s (Chen *et al.*, 1994 ▶; Hadfield & Hajdu, 1993 ▶), eventually leading to a commercial version (4DX-ray Systems AB, Uppsala, Sweden). The Cryobench laboratory of the ESRF (http://www.esrf.eu/UsersAndScience/Experiments/MX/Cryobench/) was initially opened in 1999 as a facility based around a microspec inspired by the 4DX microspec, but with a third objective added to allow, when required, the recording of fluorescence spectroscopy data (Bourgeois *et al.*, 2002 ▶). The facility was later enhanced with a mechanically more stable setup capable of recording fluorescence lifetimes (Royant *et al.*, 2007 ▶) and Raman spectra (Carpentier *et al.*, 2007 ▶). An online microspec for UV–vis absorption was developed in parallel on the structural biology beamlines of the ESRF (McGeehan *et al.*, 2009 ▶; Nanao & Ravelli, 2006 ▶). Since the inception of the Cryobench, similar apparatus has been built and installed elsewhere, notably at the Swiss Light Source in Villigen (SLSpectroLAB; http://www.psi.ch/sls/pxii/spectrolab; Owen *et al.*, 2009 ▶; Pompidor *et al.*, 2013 ▶) and at beamline X26-C at NSLS (Orville *et al.*, 2011 ▶; Stoner-Ma *et al.*, 2011 ▶). Microspecs have also been installed at the SRS in Daresbury (Ellis *et al.*, 2008 ▶), the BioCARS facility of the APS in Argonne (http://biocars.uchicago.edu/), Spring-8 (Sakai *et al.*, 2002 ▶; Shimizu *et al.*, 2013 ▶), the SSRL and Diamond Light Source (http://diamond.ac.uk/mx-home/Equipment-on-Demand/Spectroscopy.html). Additionally, a fluorescence microspec has been designed to be easily transported between home laboratories and various synchrotrons (Klink *et al.*, 2006 ▶). Microspecs can be used either during a diffraction experiment, directly mounted on the experimental setup of a synchrotron beamline (online mode), or before and/or after a diffraction experiment in a different location at the synchrotron (offline mode). The online mode is necessary when the effect of X-rays on samples is investigated. The offline mode is preferred for the time-consuming steps of characterization, experimental protocol optimization and sample preparation.

While the most common application of *ic*OS is the assessment of whether a crystallized protein is in a similar functional state as in solution (Bourgeois & Royant, 2005 ▶; Pearson *et al.*, 2004 ▶), the technique has also become of great help in monitoring the level of X-ray-induced damage (or ‘radiation damage’) occurring during crystallographic experiments on bright synchrotron beamlines (Garman, 2010 ▶). Moreover, because proteins are often active in the crystalline state, *ic*OS can be used in experiments aimed at elucidating the structures of unstable reaction-intermediate species, particularly when such species have a distinct spectroscopic signature. Crystallographic methods to determine the structure of unstable protein species, either as a function of time (reaction-intermediate state) or of X-ray dose (X-ray-sensitive state), have been named kinetic crystallography (KX; Bourgeois & Royant, 2005 ▶).

Here, we describe the third version of the ESRF Cryobench installation, summarize improvements compared with the previous version, review the various types of proteins studied and the types of experiments carried out using *ic*OS at the ESRF and comment on experimental difficulties.

## 2. Instrumental setups   

### 2.1. Offline facility (Cryobench)   

**Figure 1:**
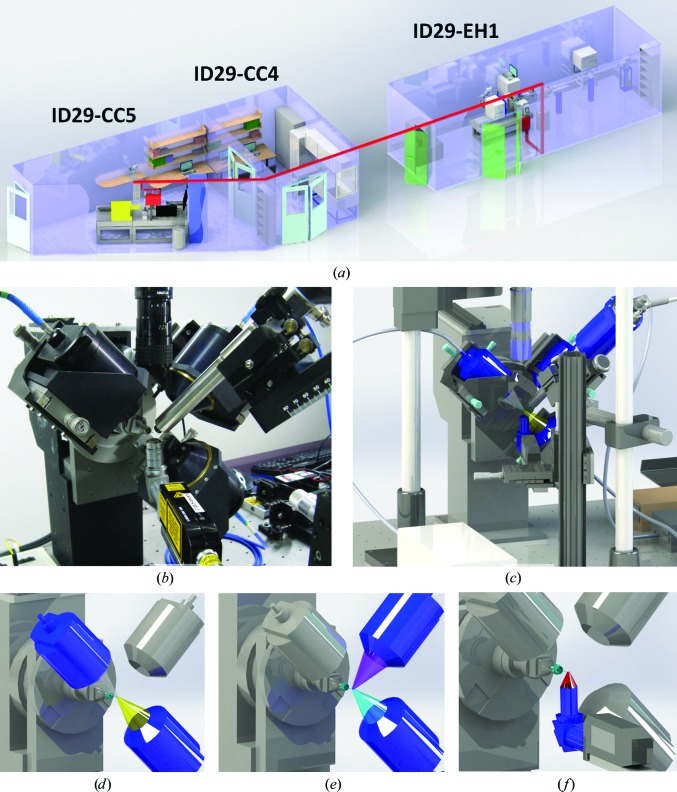
Cryobench location and setup. (*a*) The Cryobench laboratory is composed of a control room (CC4) and an experimental hutch (CC5) on beamline ID29, to the experimental hutch (EH1) of which a Raman optical fibre is connected (in red). Control rooms CC2 and CC3 have been omitted for clarity. (*b*) Photograph and (*c*) virtual representation of the experimental setup in CC5 featuring the three objectives for UV-absorption and fluorescence spectroscopy and the Raman objective (all in blue). (*d*) Objectives used for UV–vis absorption (transmission mode, 0° geometry). (*e*) Objectives used for fluorescence spectroscopy (reflection mode, 90° geometry). (*f*) Objective used for Raman spectroscopy (backscattering mode, 180° geometry).

The Cryobench is currently located next to the MAD beamline ID29 (de Sanctis *et al.*, 2012 ▶; Fig. 1 ▶ *a*). The microspectrophoto­meter setup comprises a motorized single-axis goniometer (one rotation, one translation along the same horizontal axis) holding a Huber goniometer head (Rimsting, Germany) which allows two additional degrees of translation, on which a sample, either a crystal or nanolitres of solution on a SPINE standard sample holder (Cipriani *et al.*, 2006 ▶), is mounted. Three objectives, mounted in a 90° geometry relative to each other (and a vertical camera) are 5 cm (8 cm) away from the position of the sample (Figs. 1 ▶ *b* and 1 ▶ *c*). The temperature and humidity of the sample position can be controlled with a liquid-nitrogen open-flow cooler (Cryostream Series 700, Oxford Cryosystems, Oxford, England) for temperatures ranging from 100 to 270 K or with the EMBL/ESRF dehumidifier HC1 (Sanchez-Weatherby *et al.*, 2009 ▶) for room-temperature measurements.

**Figure 2:**
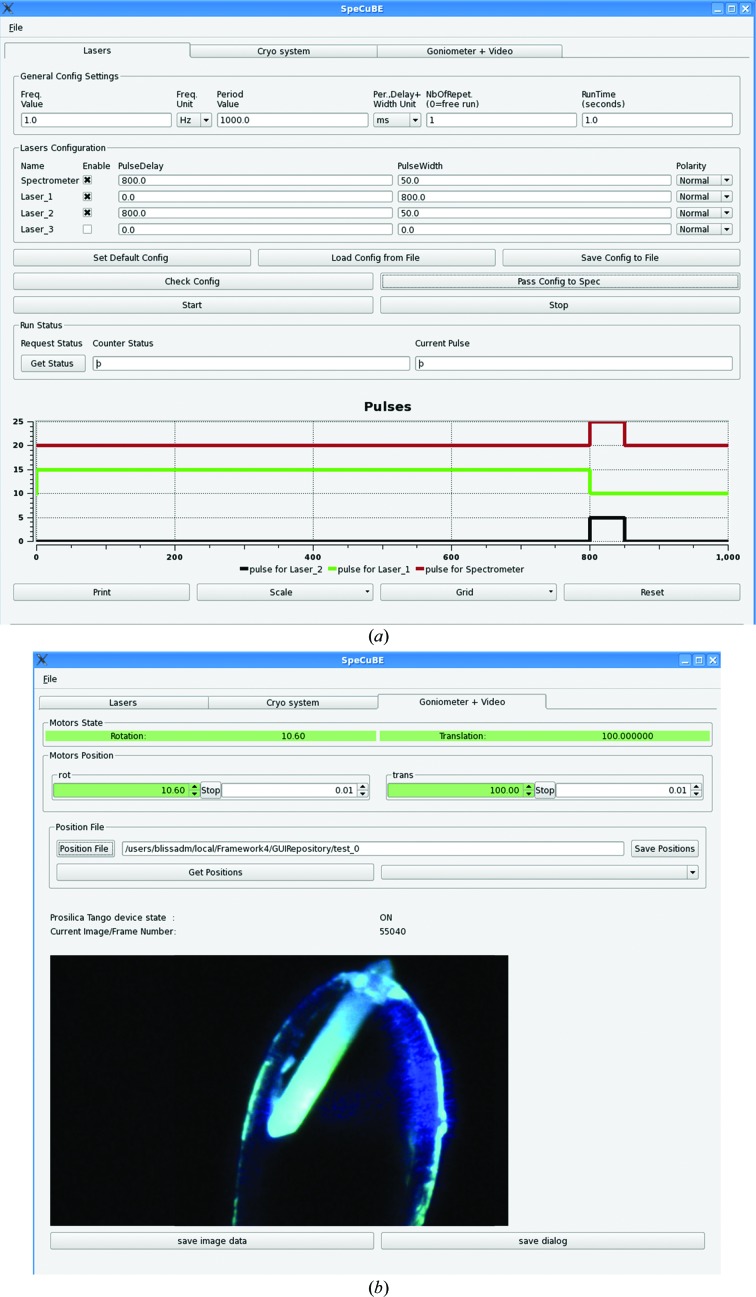
Main control window of *SpeCuBE*. (*a*) Laser tab: TTL signal generation, with up to four lines for synchronization of the spectrophotometer and one to three laser lines. (*b*) Goniometer and video tab: horizontal translation and rotation of the sample; video monitoring of the sample (live and snapshot). The featured image is that of a crystal of Cerulean under 440 nm laser excitation, exhibiting its characteristic cyan fluorescence emission.

Several technical choices have been made in order to maximize the compatibility between the environments of the Cryobench and the ESRF macromolecular crystallography beamlines. In particular, the analogue camera previously used for sample visualization has been replaced by the same digital Prosilica camera as used on the beamlines. New control modules for video acquisition, goniometer operation, laser triggering and cryosystem temperature control have been written with the BLISS FrameWork, an ESRF software-development platform, and run on Linux OS. In this way, experiment-control modules can be taken from, or ported to, the beamline-control software *MxCuBE* (Gabadinho *et al.*, 2010 ▶). All Cryobench experiment-control modules have been grouped together in a main control software called *SpeCuBE* (Fig. 2 ▶).

The UV–vis absorption mode of operation consists of using the two objectives at 180° from each other (Fig. 1 ▶ *d*). White light from a balanced deuterium–halogen lamp (Mikropack DH2000-BAL, Ocean Optics) is connected to one objective *via* an optical fibre, while a fixed-grating spectrophotometer with a CCD detector (model HR2000+ or QE65Pro, Ocean Optics) is connected to the other objective. Alignment of the spectrophotometer consists of a series of micrometric translations parallel or perpendicular to the light path that align the focal volumes of the objectives and maximize light transmission through a 30–100 µm wide pinhole (Melles-Griot) placed at the sample position. The diameter of the focal volume is one quarter of the diameter of the optical fibre (available from 50 to 1000 µm) connected to the objective.

Absorption and fluorescence emission spectra are measured with the commercial *SpectraSuite* software (Ocean Optics) running on Linux. In order to measure an absorption spectrum *A*(λ), three different spectra need to be recorded: the dark reference *I*_dark_(λ) (the spectrum in the absence of light), the light reference *I*_ref_(λ) (the lamp spectrum in the absence of a sample) and the sample spectrum *I*_sample_(λ) (the light transmitted through the sample):
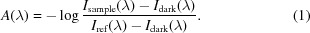



 Fluorescence emission spectra (Bourgeois *et al.*, 2002 ▶) and fluorescence lifetime histograms (Royant *et al.*, 2007 ▶) are measured using laser lines as excitation light with two objectives at 90° from each other in order to minimize excitation laser scattering to the CCD detector (Fig. 1 ▶ *e*). Because fluorophores are usually photoactivatable or photobleachable, laser irradiation needs to be kept to a minimum by synchronizing laser triggering pulses (typically of milliseconds to hundreds of milliseconds in duration) with the recording of spectra *via* TTL pulses generated by an ESRF-developed board (OPIOM type). Laser lines can be chosen from more than ten typical wavelengths ranging from 266 nm (deep UV) to 671 nm (far red), with a maximum power of 10–100 mW at the sample position.

**Figure 3:**
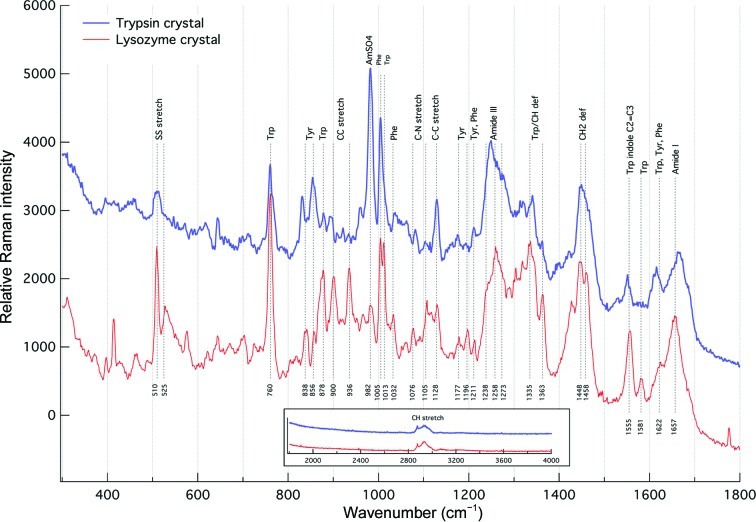
Raman spectra of two protein crystals: trypsin (blue) and lysozyme (red). Spectra were recorded at 100 K for 100 s with a 785 nm laser. Band assignment was performed using previously published work (Barth & Zscherp, 2002 ▶; Carpentier *et al.*, 2007 ▶; Jacob *et al.*, 1998 ▶; Krimm & Bandekar, 1986 ▶; Peticolas, 1995 ▶).

Raman spectra are measured in backscattering mode using a Renishaw InVia spectrometer (Carpentier *et al.*, 2007 ▶), the probe of which has been modified by the addition of a 45° mirror inserted before the microscope objective (Fig. 1 ▶ *f*). Spectra are recorded with the commercial software *Wire*3 running on a Windows PC. Raman spectra exhibit many more peaks than UV–vis absorption or fluorescence emission spectra; hence, their interpretation relies on a comparison with reference spectra taken from other protein crystals (Fig. 3 ▶).

### 2.2. Online UV–vis absorption microspectrophotometer (‘online microspec’)   

The first apparatus used to measure UV–vis absorption spectra directly on macromolecular crystallography beamlines was designed in the context of a collaborative effort between the University of Oxford, the EMBL Grenoble and the ESRF (Nanao & Ravelli, 2006 ▶). A more compact version was then developed by the EMBL and commercialized by MAATEL (Voreppe, France; McGeehan *et al.*, 2009 ▶). This apparatus was designed to monitor the buildup and decay of various X-ray-induced phenomena such as solvated electrons or disulfide-bond radicals with spectroscopic signatures in the UV–visible range (Weik *et al.*, 2002 ▶; McGeehan *et al.*, 2009 ▶). It can also be used to identify X-ray-excited optical luminescence (XEOL) signals from protein crystals, which could serve as another tool to study specific radiation damage (Owen *et al.*, 2012 ▶). The online microspec has since been used to monitor the X-ray-induced spectroscopic changes of many coloured proteins and in the design of optimal strategies for diffraction data collection (*i.e.* the collection of composite data sets from one or several crystals; Adam *et al.*, 2004 ▶; Berglund *et al.*, 2002 ▶; Bourgeois *et al.*, 2009 ▶; Pearson *et al.*, 2004 ▶) in order to elucidate crystal structures unaffected by radiation damage; for instance, metalloproteins containing metal centres in the correct oxidation state.

The fluorescence option was implemented by adapting the Cryobench fluorescence setup (Royant *et al.*, 2007 ▶). A National Instruments board was added to a laptop station which can generate series of TTL signals (at 0.1–10 Hz repetition rates) that are used to synchronize laser irradiation and spectrophotometer recording *via* a *LabVIEW* (National Instruments) application running on a Windows laptop. Because there is no third objective in this setup, either a split fibre has to be connected to one objective (one fibre for excitation light; the other one being connected to the spectrophotometer) or the laser and the spectrophotometer are connected to the two opposing objectives. In the latter case, a filter rejecting the laser line is required to prevent saturation of and damage to the CCD detector.

### 2.3. Online Raman spectrometer   

Online Raman spectroscopy has been made possible on beamlines by successive rounds of improvement. The original experiments were performed by connecting the Raman spectrometer, mounted on a rolling table, to the beamline experimental hutch using a 20 m fibre. The experimental hutch ID29-EH1 is now permanently connected to the Raman spectrometer of the Cryobench *via* a set of 50 m optical fibres (red trace in Fig. 1 ▶ *a*), and a Raman head support has been designed to fit the MD2 diffractometer (Maatel, Voreppe, France). The support itself will be described elsewhere. In the near future, the Cryobench will be similarly connected to an experimental hutch of the new MASSIF suite of endstations situated on the straight section ID30 next to ID29 (Theveneau *et al.*, 2013 ▶). The use of Raman spectroscopy in conjunction with X-ray crystallography has been named ‘Raman-assisted X-ray crystallography’ (RaX) and its use has already been extensively reviewed (Bourgeois *et al.*, 2009 ▶; McGeehan *et al.*, 2011 ▶).

## 3. Experimental considerations   

Solution spectroscopy usually imposes limits on the concentration of the sample: for UV–vis absorption, one aims at limiting the optical density (OD) to approximately 1.0 (above this limit particle–particle interactions affect light absorption and the OD is no longer a linear function of concentration). For fluorescence spectroscopy, it is desirable to limit the concentration of the sample to a maximum OD of 0.05 to minimize absorption of the fluorescence signal by surrounding molecules. *ic*OS differs from solution spectroscopy in the sense that the concentration cannot be precisely adjusted, apart from choosing crystals of various thicknesses or focusing light at the crystal edges (where they are thinner). Moreover, protein crystals are highly concentrated in chromophores and other chemical groups with a spectroscopic signature. As a consequence, the guidelines for carrying out spectroscopy on crystals are markedly different from those for similar experiments on solutions. Several considerations must therefore be taken into account and are described below.

### 3.1. Alignment issues   

**Figure 4:**
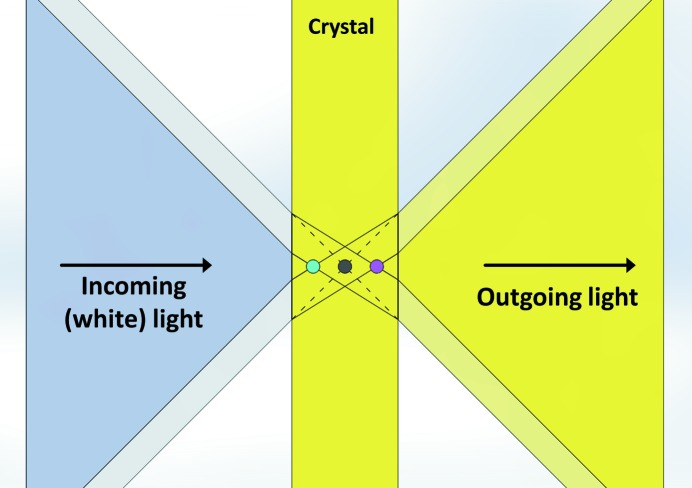
Principle of light defocusing by a crystal. The focal volume of both objectives in air (*n* = 1.00) is represented by the black dot. When a crystal is present, each focal volume is translated to either the purple dot (white light objective) or the cyan dot (spectrophotometer objective) owing to refraction of light within the crystal (*n* > 1.00).

The most important issue to consider when aligning a sample is the fact that the optical path has to cross various materials with distinct refraction indices: air (*n* = 1.00), mother liquor [1.33 (pure water) ≤ *n* ≤ 1.5 for salts and PEGs] and crystal (*n* ≃ 1.34 at high protein concentrations; Cole *et al.*, 1995 ▶). The alignment of a two-objective microspec consists of matching, in three dimensions, the two focal volumes in the absence of a sample (*i.e.* when the whole optical path is in air). If the sample is large or if the mother liquor has not been removed and forms a bulk around the sample, the focal volumes will be significantly displaced. Fig. 4 ▶ shows a simple situation in which the beam path is perpendicular to the surface of a plate-shaped crystal. The two objectives have been aligned in air with their focal volumes matched at the position of the black dot. Insertion of the protein crystal with a higher refractive index at the sample position shifts both focal volumes in opposite directions. As a consequence of the mismatch between the focal volumes, the transmitted light is not optimally focused onto the entrance of the optical fibre leading to the spectrophotometer, thus decreasing the signal-to-noise ratio of the measurement. In other words, the reference light signal *I*_ref_ (equation 1) recorded in the absence of a sample differs from the hypothetical *I*_ref_ that would be recorded in the presence of a noncoloured sample of the same shape. However, the resulting absorption spectrum only differs from the hypothetical spectrum by an offset of the baseline, which can be easily corrected for. This situation is aggravated by increases in the size of the sample and the tilt of its position *versus* the light axis. The effect is even more severe for nonplanar crystalline samples, for example a crystal in a frozen drop of liquid, which results in a lens effect. In this case not only are the focal volumes displaced, but the direction of the transmitted light is also altered. A partial remedy for this issue consists of translating the second objective with the sample in place while monitoring the increase in the collected light until a maximum is achieved.

### 3.2. Self-absorption effect   

The effect of strongly absorbing crystals on the shape of fluorescence emission spectra has been reported for light-harvesting complex II (LHCII; Barros *et al.*, 2009 ▶). LHCII is a photosynthetic protein that contains many different coloured cofactors (carotenoids and chlorophylls) and is one of the proteins with the most coloured cofactors per amino-acid residue. Consequently, even very thin LHCII crystals (10 µm) have an OD above 2.5 and act as bandpass filters in the blue and red regions of the spectra.

**Figure 5:**
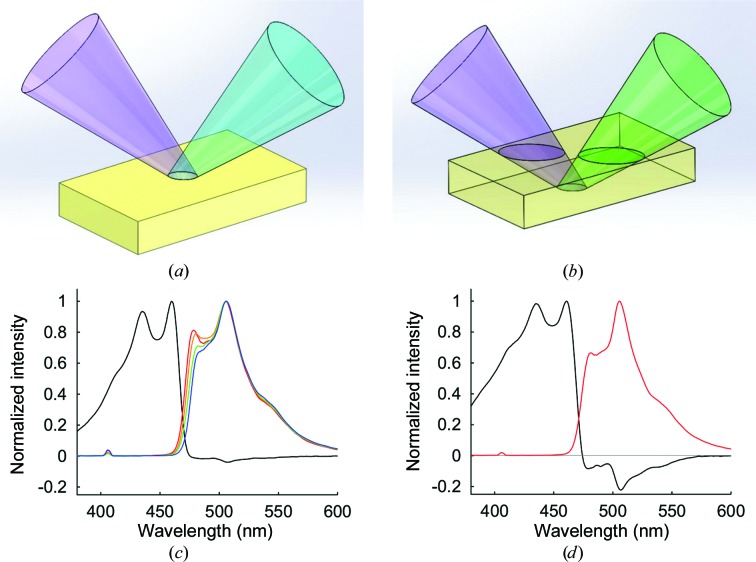
Optical artefacts in a coloured crystal. (*a*) Fluorescence excitation at the surface of a crystal. (*b*) Fluorescence excitation at the opposing face, with the emission light path traversing the whole thickness of the crystal. (*c*) Absorption (black) and emission spectra [red, geometry described in (*a*); blue, geometry described in (*b*); orange and green, intermediate situations] of a crystal of the cyan fluorescent protein Cerulean (Lelimousin *et al.*, 2009 ▶). (*d*) Absorption spectrum (black) of a crystal of Cerulean exhibiting a negative optical density around the maximum fluorescence emission (red).

This so-called ‘self-absorption’ (or ‘inner filtering’) is modulated by the geometry of sample illumination. If the excitation and emission objectives are focused on the top surface of a crystal of the fluorescent protein Cerulean (Lelimousin *et al.*, 2009 ▶; Fig. 5 ▶ *a*), one is able to measure the genuine emission spectrum of the protein (the red trace in Fig. 5 ▶ *c*). However, if one focuses on the bottom surface, such that both the excitation and the emission light travels through the crystal (Fig. 5 ▶ *b*), the significant OD of the crystal (see the absorption spectrum depicted in black in Fig. 5 ▶ *c*) will absorb most of the low-wavelength emitted photons, resulting in an apparent red shift of the left emission peak (orange, green and blue traces in Fig. 5 ▶ *c*).

### 3.3. Negative absorption owing to fluorescence   

During the recording of UV–vis absorption spectra from fluorescent samples, the incident white light also produces some detectable fluorescence. Depending on the geometry of the two focal volumes (see above), a significant amount of this unwanted fluorescence will be recorded by the collecting objective, which leads to a dip in the resulting absorption spectrum (black trace in Fig. 5 ▶ *d*) in the region of maximum fluorescence in the emission spectrum (the red trace in Fig. 5 ▶ *d*), formally constituting a negative absorption signal.

## 4. Biological systems studied at the Cryobench   

**Figure 6:**
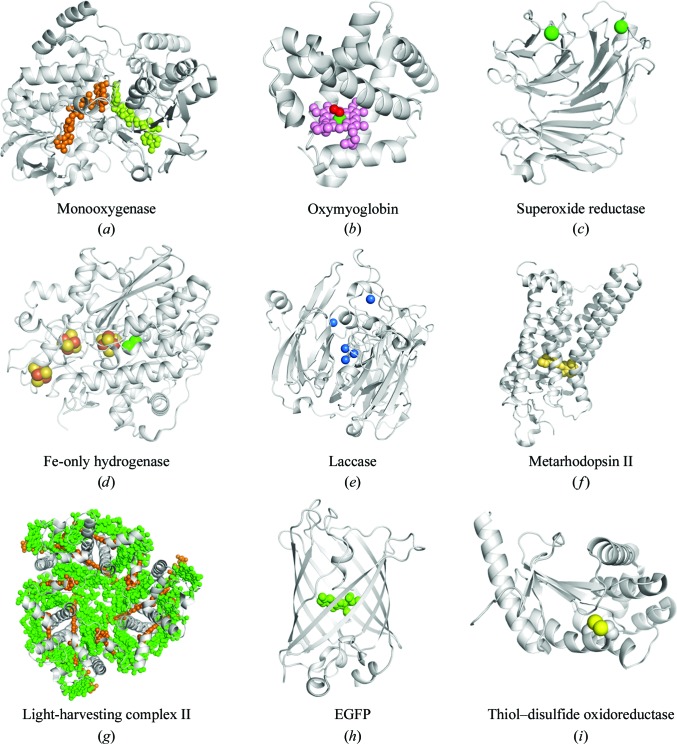
Structures of representative proteins studied at the Cryobench shown with their spectroscopically active groups coloured. (*a*) Monooxygenase: NADPH and FAD (PDB entry 2ylr; Orru *et al.*, 2011 ▶). (*b*) Oxymyoglobin: haem (PDB entry 2vlx; Hersleth *et al.*, 2008 ▶). (*c*) Superoxide reductase: nonhaem iron (PDB entry 1vzi; Adam *et al.*, 2004 ▶). (*d*) Iron-only hydrogenase: Fe–S clusters (PDB entry 1hfe; Nicolet *et al.*, 2001 ▶). (*e*) Laccase: copper (PDB entry 2x88; Bento *et al.*, 2010 ▶). (*f*) Metarhodopsin II: retinal (PDB entry 3pxo; Choe *et al.*, 2011 ▶). (*g*) Light-harvesting complex II: chlorophylls and carotenoids (PDB entry 2bhw; Barros *et al.*, 2009 ▶; Standfuss *et al.*, 2005 ▶). (*h*) Enhanced green fluorescent protein: *p*-­hydroxybenzylidene-imidazolinone (PDB entry 2y0g; Royant & Noirclerc-Savoye, 2011 ▶). (*i*) Thiol–disulfide oxidoreductase: disulfide bond (PDB entry 3hz8; Lafaye *et al.*, 2009 ▶).

Systems studied using Cryobench setups include proteins with coloured cofactors (FAD and NADPH), metalloproteins of various kinds (containing copper, nonhaem iron, haem iron or iron–sulfur clusters), photoactive proteins with isomerizable chromophores (retinal and bilins), fluorescent proteins homologous to GFP and proteins containing disulfide bridges (Figs. 6 ▶ and 7 ▶). A selection of representative publications is listed in Table 1 ▶, allowing rapid identification of the type of application, the operation mode (offline/online), the spectroscopic technique used, the nature of the sample (crystal/solution) and the temperature range.

### 4.1. Enzymes with coloured cofactors   

Many enzymes rely on cofactors that absorb in the near-UV/blue part of the light spectrum. Usually, the light-absorption properties of the cofactor are not instrumental to the catalytic mechanism. Measurement of the fluorescence emission spectrum from crystals of NADH-containing malate dehydrogenase was one of the first experiments carried out at the Cryobench, identifying the redox state of NADH in the crystal (Bourgeois *et al.*, 2002 ▶). Crystals of a Baeyer–Villiger monooxygenase, containing NADPH and FAD as the electron donor and oxygen acceptor, respectively, were studied both at the Cryobench and with the online microspec in order to monitor the reversible oxidation of the flavin by air (at room temperature) (Figs. 6 ▶ *a* and 7 ▶ *a*) or reduction by either dithionite or X-rays (Orru *et al.*, 2011 ▶). Finally, a vitamin B_6_-dependent phosphoserine aminotransferase has been shown using online UV–vis absorption spectroscopy to be sensitive to X-rays at the location of its lysine–pyridoxal-5′-phosphate (lysine–PLP) Schiff-base linkage (Dubnovitsky *et al.*, 2005 ▶).

The only two known enzymes for which light-induced activation of cofactors is necessary for the catalysed reaction have both been studied at the Cryobench. DNA photolyase uses two cofactors, the flavin FAD and a light-harvesting chromophore which is either 8-hydroxy-5-deazaflavin or 5,10-methenyltetrahydrofolate, to repair DNA lesions caused by UV radiation in bacteria. Online UV–vis absorption spectroscopy was used to monitor light-induced or X-ray-induced reduction of the cofactors (Kiontke *et al.*, 2011 ▶; Kort, Komori *et al.*, 2004 ▶; Moldt *et al.*, 2009 ▶). Light-dependent protochlorophyllide oxidoreductase (POR) catalyses the penultimate step of chlorophyll biosynthesis. Temperature-dependent fluorescence spectroscopy was used on solution samples to study the influence of solvent dynamics on the formation of the first two reaction-intermediate states (Durin *et al.*, 2009 ▶).

### 4.2. Metalloproteins   

Haem proteins, with an Fe atom bound to a porphyrin ring, are gas or electron transporters or enzymes. Early offline studies attempted to establish redox states before and after X-ray data collection (Dias *et al.*, 2004 ▶; Hersleth *et al.*, 2008 ▶; Marcaida *et al.*, 2006 ▶; Williams *et al.*, 2006 ▶) and showed their high X-ray susceptibility, the explanation for which must reside in their redox activity (Figs. 6 ▶ *b* and 7 ▶ *b*). Later studies used the online microspec to monitor the rate of haem photoreduction, a phenomenon which was eventually used to mimic the physiological redox reaction (Gumiero *et al.*, 2011 ▶; Hersleth & Andersson, 2011 ▶; Macedo *et al.*, 2009 ▶). Besides these more complex experiments, offline studies were used to characterize the functional state of haems in protein crystals (Rajagopal *et al.*, 2011 ▶; Wahlgren *et al.*, 2012 ▶).

Some nonhaem iron enzymes have their catalytic iron coordinated by at least four amino-acid residues of the protein. The superoxide reductase (SOR) from the microaerophilic bacterium *Desulfoarculus baarsii* falls into this category. SOR acts as a defence against oxidative stress and presents the advantage over superoxide dismutase of not releasing dioxygen upon superoxide reduction. A composite data-collection strategy (Berglund *et al.*, 2002 ▶; Pearson *et al.*, 2004 ▶) taking advantage of online UV–vis microspectrophotometry allowed comparison of its reduced and oxidized states (Adam *et al.*, 2004 ▶). Subsequently, in order to prove the presence in the crystals of a peroxo adduct on the iron with an end-on geometry, Raman spectroscopy was used directly on crystals soaked with either H_2_^16^O_2_ or H_2_^18^O_2_ to show the presence of an Fe—O covalent bond (Katona *et al.*, 2007 ▶; Figs. 6 ▶ *c* and 7 ▶ *c*). Other nonhaem iron proteins contain geometric arrangements of Fe and S atoms: iron–sulfur cluster proteins. UV–vis absorption spectroscopy has been used to investigate the redox state of Fe–S clusters (Aragão *et al.*, 2003 ▶; Sainz *et al.*, 2006 ▶) or of catalytic Fe atoms (Nicolet *et al.*, 2001 ▶; Figs. 6 ▶ *d* and 7 ▶ *d*).

The X-ray-induced photoreduction of the mononuclear copper electron-transfer protein azurin and of multi-copper oxidases (MCOs) has been investigated using online microspectrophotometry, either directly in crystals (Ferraroni *et al.*, 2012 ▶; Macedo *et al.*, 2009 ▶; Figs. 6 ▶ *e* and 7 ▶ *e*) or indirectly by monitoring the buildup of solvated electrons in protein solutions (De la Mora *et al.*, 2012 ▶). Another MCO had its spectroscopic signature in crystals compared with that in solution (Bento *et al.*, 2010 ▶). Finally, crystals of CnrX, the metal-sensing domain of a membrane protein complex implicated in bacterial resistance to nickel and cobalt, was shown to bind one divalent cobalt cation in a high-spin state by UV–vis absorption spectroscopy (Trepreau *et al.*, 2011 ▶).

### 4.3. Photoactive proteins   

Photoactive proteins contain chromophores, the light-induced isomerization of which triggers structural rearrangements of the protein leading to the fulfilment of its function.

One such family of proteins studied at the Cryobench is that of the retinal proteins, which exhibit very distinct functions in various organisms. One early intermediate state of bacterio­rhodopsin, a proton pump from the halophilic archaeon *Halobacterium salinarum*, has been characterized using either excitation by a green (Royant *et al.*, 2000 ▶) or a red laser (Edman *et al.*, 2004 ▶) at low temperature. A similar study using a blue-cyan laser was performed on sensory rhodopsin II, an archaeal photoreceptor implicated in negative phototaxis (Edman *et al.*, 2002 ▶). Various intermediate states in the photocycle of bovine rhodopsin, the protein responsible for vision, were similarly characterized upon excitation at low temperature (Okada *et al.*, 2002 ▶) and also after reconstitution with the isomerized chromophore at room temperature (Choe *et al.*, 2011 ▶) (Figs. 6 ▶ *f* and 7 ▶ *f*).

Photoactive yellow protein (PYP), thought to be implicated in bacterial phototaxis, has been the topic of compelling structural studies using Laue crystallography of intermediate states on the picosecond-to-second timescale (Jung *et al.*, 2013 ▶; Ihee *et al.*, 2005 ▶). PYP was studied with low-temperature monochromatic crystallography, using UV–vis light absorption as a complementary method to probe the altered kinetics of the photocycle in the crystalline state compared with those observed in solution (Kort, Hellingwerf *et al.*, 2004 ▶; Kort *et al.*, 2003 ▶).

Phytochromes are red/far-red photochromic biliprotein photoreceptors which regulate many light-affected processes in plants (notably the germination of seeds and flowering) and certain bacteria and fungi. Isomerization of their bilin chromophore leads to a conformational change which triggers downstream signal transduction. UV–vis absorption and Raman spectroscopy were used to compare the crystalline protein with its solution state and showed that in the former photoconversion to the far-red-absorbing state was significantly hampered but was not completely inhibited (Anders *et al.*, 2013 ▶; Essen *et al.*, 2008 ▶; Mailliet *et al.*, 2009 ▶, 2011 ▶). This suggests that phytochromes may be functional in the crystalline state but that crystal contacts prevent the large-scale domain movements necessary for full functionality.

Photosynthetic proteins are a special case of photoactive proteins where chromophore isomerization is not required for function. Their functional role is architectural: they hold in place a multitude of chromophores covering the whole visible-light spectrum in order to harvest sunlight optimally and transfer energy quanta to the active site of photosystems. Crystals of such a protein, light-harvesting complex II from pea, were thoroughly investigated by UV–vis absorption and emission fluorescence spectroscopies and fluorescent lifetime measurements to show that the protein was in the active, light-transmitting state (Barros *et al.*, 2009 ▶; Figs. 6 ▶ *g* and 7 ▶ *g*). α-Phycoerythrocyanin (PEC) is a bilin photoreceptor that is part of the light-harvesting complex, the phycobilisome, of certain cyanobacteria. Phycobilisomes absorb light and transfer its energy to the photosynthetic reaction centres, acting as a light-harvesting antenna. Phycoviolobilin, the chromophore of PEC, isomerizes between two stable configurations upon light irradiation. UV–vis absorption was used to monitor the efficiency of photoconversion, and the structures of the two isomeric forms were eventually obtained to provide the structural basis of PEC photochemistry (Schmidt *et al.*, 2007 ▶).

### 4.4. Fluorescent proteins   

While it was rapidly realised that the chromophores of fluorescent proteins, as well as the surrounding residues, were particularly sensitive to radiation damage by X-rays (Adam *et al.*, 2009 ▶; Royant & Noirclerc-Savoye, 2011 ▶; Figs. 6 ▶ *h* and 7 ▶ *h*) and thus that special care is required during data collection and structural analysis, the Cryobench has become an essential instrument to study the structure and function of proteins homologous to green fluorescent protein (GFP; Tsien, 1998 ▶). The most common application of the Cryobench is to compare the spectroscopic signatures of different proteins in crystals with those in solution. While these are generally very similar (Carpentier *et al.*, 2009 ▶; Goedhart *et al.*, 2012 ▶; Lelimousin *et al.*, 2009 ▶; Violot *et al.*, 2009 ▶; von Stetten, Noirclerc-Savoye *et al.*, 2012 ▶), fluorescence lifetimes are shorter in crystals, which is related to the higher refractive index of the crystal mother liquor (Royant *et al.*, 2007 ▶; Suhling *et al.*, 2002 ▶), and the fact that diffusion of small molecules close to the chromophore can induce subtle rearrangements of side chains and subsequent displacement of absorption and emission peaks (von Stetten, Batot *et al.*, 2012 ▶). Moreover, the Cryobench is adapted to the elucidation of various reversible or irreversible mechanisms of fluorescent proteins: photoactivation, photobleaching and photoswitching. These studies take advantage of the various available laser lines available at the Cryobench as well as the possibility of adjusting the temperature at which measurements are made (Adam *et al.*, 2008 ▶; de Rosny & Carpentier, 2012 ▶; Duan *et al.*, 2013 ▶; Faro *et al.*, 2010 ▶, 2011 ▶).

### 4.5. Noncoloured proteins or DNA   

All of the examples given above highlight studies of proteins that are coloured. Optical spectroscopy can, in some cases, also be applied to noncoloured samples which contain specific chemical bonds. Disulfide bonds bridging two cysteines result in a distinct Raman stretching mode because of the greater mass of S atoms and their relative scarcity within proteins. Raman spectroscopy has thus been used to study the presence, or absence, of disulfide bonds in the active sites of various mutants of the thiol–disulfide oxidoreductase DsbA (Lafaye *et al.*, 2009 ▶; Figs. 6 ▶ *i* and 7 ▶ *i*). Disulfide bonds are known for their X-ray sensitivity (Weik *et al.*, 2000 ▶). UV–vis absorption can be used to identify the presence of radiation damage by monitoring the formation of a disulfide radical, an intermediate state in bond reduction, in various protein crystals (McGeehan *et al.*, 2009 ▶; Murray & Garman, 2002 ▶; Weik *et al.*, 2002 ▶) and online Raman spectroscopy can be used to monitor the kinetics of X-ray-induced disulfide-bond photoreduction (Carpentier *et al.*, 2010 ▶; McGeehan *et al.*, 2011 ▶). Similarly, X-ray-induced debromination of brominated DNA crystals can also be monitored using Raman spectroscopy (McGeehan *et al.*, 2007 ▶).

## 5. Methodological applications   

As well as being crucial in providing complementary information in the study of the crystal structures of certain biological macromolecules, the Cryobench can be used in applications of more general use. Heavy-atom soaking can be monitored if it produces a species with a specific spectroscopic signature (*e.g.* a mercury–sulfur covalent bond), thus allowing a precise determination of optimal (at least in terms of heavy-atom binding) soaking times (Carpentier *et al.*, 2007 ▶); the exposure of crystals to UV light can be used to locate crystals in loops by their intrinsic fluorescence (Jacquamet *et al.*, 2004 ▶; Vernede *et al.*, 2006 ▶) or to deliberately induce radiation damage to crystals, which can then be exploited to provide phase information for crystal structure solution *via* UV-RIP (Nanao & Ravelli, 2006 ▶); the pH in a crystal can be directly determined by soaking samples in an exogenous pH-sensitive fluorophore (Bourgeois *et al.*, 2002 ▶; Fioravanti *et al.*, 2003 ▶). Under favourable circumstances, optical spectroscopy can also be used to identify unknown ligands that are found in electron-density maps to be bound to a protein. Examples here include the use of Raman spectroscopy to unambiguously prove the binding of a nitrate ion to xylose isomerase (Carpentier *et al.*, 2007 ▶) and the identification of chlorophyll *a* and carotenoids, in substoichiometric amounts, in crystals of the c-ring of a proton-coupled F_1_F_o_ ATP synthase (Pogoryelov *et al.*, 2009 ▶).

The Cryobench is also an indispensible tool for kinetic crystallography (KX) experiments based on the use of caged compounds. Synchronization of de-caging can be achieved with an actinic light. In this regard, noncoloured proteins can be made coloured in the near-UV range by chemically grafting a photolabile group onto either a substrate (*e.g.* deoxy­thymidine monophosphate), cofactor (*e.g.* adenosine triphos­phate or dioxygen) or product [*e.g.* (arseno)choline] of the protein (Colletier *et al.*, 2007 ▶; Howard-Jones *et al.*, 2009 ▶; Specht *et al.*, 2001 ▶; Ursby *et al.*, 2002 ▶). Less expectedly, the Cryobench can also be used in experiments that use KX to study the catalysis of inorganic complexes. Because crystals of small molecules allow very little movement of the molecules that they contain, KX experiments on such systems are difficult to perform. However, in an elegant approach, reaction-intermediate states of an inorganic iron complex, as monitored using UV–vis absorption and Raman spectroscopies, were trapped using crystals of a protein with a large cavity and their three-dimensional structures were solved (Cavazza *et al.*, 2010 ▶).

A final advantage of the Cryobench is that the temperature at which measurements are carried out can be varied. In order to prepare KX experiments, temperature-derivative fluorescence, or absorbance, microspectrophotometry (TDFM/TDAM) has been developed to allow the monitoring of solvent phase transitions in protein crystals (Weik *et al.*, 2004 ▶) and, in protein solutions, to determine whether the correlation between solvent and protein motions is necessary for the formation of reaction-intermediate states (Durin *et al.*, 2009 ▶).

## 6. Perspectives   

The development of the ESRF Cryobench facility is a continuous effort that endeavours to keep pace with progress on the macromolecular crystallography beamlines situated nearby. Two main aspects can be outlined: automation and online spectroscopy. More and more samples are now brought or sent to synchrotrons for diffraction experiments. Complementing these experiments with optical spectroscopic analysis thus requires automation of sample mounting and both positional and angular centring in both the offline and online setups. The current upgrade of beamlines requires adaptation of optical setups to newly designed experimental hutches. To begin with, an improved Raman setup has been installed on ID29 and will eventually be duplicated on one of the new MASSIF structural biology beamlines (Theveneau *et al.*, 2013 ▶).

## Figures and Tables

**Figure 7:**
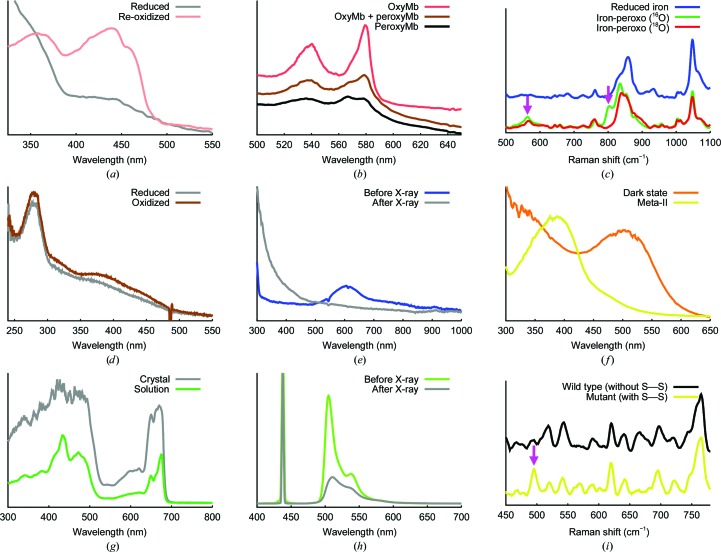
Spectroscopic data corresponding to the proteins represented in Fig. 6 ▶. (*a*, *d*) Comparison of reduced and oxidized proteins. (*b*, *e*) Photoreduction monitored by UV-vis absorption. (*c*) Buildup of a peroxo intermediate demonstrated by Raman spectroscopy using isotopically labelled hydrogen peroxide. The isotopic effect is indicated by purple arrows. (*f*) Comparison of the dark and meta-II intermediate states of bovine rhodopsin. (*g*) Comparison of absorption spectra in solution and crystalline states. (*h*) Photobleaching of a fluorescent protein monitored by fluorescence spectroscopy. (*i*) The presence of a disulfide bond probed by Raman spectroscopy. The position of the disulfide-bridge stretching mode is indicated by an arrow.

**Table 1:** Highlighted papers featuring experimental results obtained using *ic*OS at the Cryobench and/or on macromolecular crystallography beamlines Abbreviations: FC, functional characterization; RD, radiation damage; KX, kinetic crystallography; Off, offline operation; On, online operation; Abs, UVvis absorption; Fluo, fluorescence; Lifet, fluorescence lifetime measurement; Act, actinic light; Sol, solution; Cryst, crystal; Cryo, cryogenic temperature; RT, room temperature.

	Application			Mode		Technique					Sample		Temperature	
Reference	FC	RD	KX	Off	On	Abs	Fluo	Lifet	Raman	Act	Sol	Cryst	Cryo	RT
Royant *et al.* (2000 ▶)														
Nicolet *et al.* (2001 ▶)														
Okada *et al.* (2002 ▶)														
Adam *et al.* (2004 ▶)														
Kort, Hellingwerf *et al.* (2004 ▶)														
Colletier *et al.* (2007 ▶)														
Katona *et al.* (2007 ▶)														
Essen *et al.* (2008 ▶)														
Adam *et al.* (2009 ▶)														
Barros *et al.* (2009 ▶)														
Carpentier *et al.* (2010 ▶)														
Cavazza *et al.* (2010 ▶)														
Choe *et al.* (2011 ▶)														
Faro *et al.* (2011 ▶)														
Gumiero *et al.* (2011 ▶)														
Kiontke *et al.* (2011 ▶)														
Orru *et al.* (2011 ▶)														
Goedhart *et al.* (2012 ▶)														
de Rosny Carpentier (2012 ▶)														
von Stetten, Batot *et al.* (2012 ▶)														
Anders *et al.* (2013 ▶)														
